# A cohort study comparing internal fixation for undisplaced versus hip arthroplasty for displaced femoral neck fracture in the elderly: a pilot study for a clinical trial

**DOI:** 10.1186/s40814-020-00642-w

**Published:** 2020-07-11

**Authors:** Sebastian Mukka, Pontus Sjöholm, Athir Aziz, Thomas Eisler, Bakir Kadum, Ferid Krupic, Per Morberg, Arkan Sayed-Noor

**Affiliations:** 1 Department of Surgical and Perioperative Sciences, Umeå University, Umeå, Sweden; 2 Department of clinical sciences at Danderyds Hospital, Karolinska Institutet, Stockholm, Sweden; 3 Department of Biomedical and Clinical Sciences (BKV), Linköping University, Linköping, Sweden; 4 Institute of Clinical Sciences, The Sahlgrenska Academy, University of Gothenburg, Gothenburg, Sweden

**Keywords:** Hip fractures, Undisplaced, Displaced, Internal fixation, Arthroplasty, Reoperation

## Abstract

**Introduction:**

The literature regarding undisplaced femoral neck fractures (FNF) is sparse. The aim of this prospective feasibility study is to compare the clinical outcome after undisplaced FNF treated with internal fixation (IF) and displaced FNF treated with hip arthroplasty. We hypothesized that hip arthroplasty would give a lower incidence of reoperations.

**Methods:**

A total of 235 patients were included with a median age of 84 years (range 65–99). A consecutive series of 65 patients with undisplaced FNF were treated with IF, and 170 patients with displaced FNF were treated with either a total hip arthroplasty or a hemiarthroplasty. Follow-up interviews were conducted at 1 year using the Harris Hip Score (HHS), WOMAC, and pain numeric rating scale (PNRS). The minimum follow-up time was 22 months. There was no difference in baseline data between the groups.

**Results:**

Nineteen (8%) hips required reoperation at least once at a mean of 6 months (range 0–35). The rate of reoperation was higher in the IF group compared to the hip arthroplasty group (13.8% vs. 5.9%, 95% CI 0.9–6.4). The overall 1-year and 2-year mortality was 28% and 40%, respectively, with no difference between the groups. The most common reasons for reoperations in the IF group were non-union and avascular necrosis, and 6 patients were treated with hip or excision arthroplasty. In the arthroplasty group, the most common indications were deep infection and dislocation. We did not find any differences between the groups in terms of HHS, WOMAC, and PNRS.

**Conclusions:**

In this feasibility study, we found no differences in patient-reported outcomes between the groups although IF required a higher rate of reoperations. Further randomized trials are needed to establish the optimal treatment of undisplaced FNF in the elderly.

**Trial registration:**

ClinicalTrial.org, NCT03392285. Retrospectively registered on 5 February 2018.

## Introduction

The incidence and healthcare burden of femoral neck fractures (FNFs) are forecasted to increase in the future due to an aging population [1]. FNFs are classified as undisplaced or displaced. Doubts have been raised regarding the results of the internal fixation of minimally displaced FNF [2]. In elderly patients, reoperation rates ranging from 8 to 19% have been reported [2–5]. The alternative treatment using hip arthroplasty is controversial. However, modern hip arthroplasty provides a hip that provides immediate unrestricted mobilization and may lower rates of reoperation, despite the increased risk for postoperative infection [6].

There is a need for large national or international, multicenter randomized clinical trials to improve the care of hip fracture patients [7–9]. Displaced femoral neck fracture in the elderly is mainly treated with hip arthroplasty in contrast to minimally displaced or undisplaced fracture which is mainly treated with internal fixation. This allows for a comparative study in order to extrapolate the results of a displaced femoral neck fracture population treated with arthroplasty to the possible results of arthroplasty for minimally displaced or undisplaced fracture.

### Rationale for a pilot study

The aim of the present prospective pilot study is to compare the clinical outcome after undisplaced and minimally displaced FNF treated with internal fixation (IF) and displaced FNF treated with hip arthroplasty, in order to provide baseline data for a sample size calculations for a large national registry-based randomized controlled study [10].

### Specific pilot objectives

The objectives are to evaluate the risk for reoperation, patient-reported outcome, and mortality after treatment of FNF with either hip arthroplasty or IF and to be able to perform a sample size calculation for a randomized controlled trial.

## Patients and methods

### Study setting

The study was conducted between February 2012 and October 2015 at the Department of Orthopaedics, Sundsvall Hospital, Sweden, which is an emergency hospital, affiliated to Umeå University, and provides medical care to a catchment area of approximately 160,000.

### Patients

We included a consecutive series of all patients aged 65 years and above who were treated for a minimally displaced or undisplaced (Garden I or II) FNF with IF or for a displaced FNF with a primary hip arthroplasty by a direct lateral approach. Either a consultant orthopedic surgeon or a registrar performed all operations on the day of admission or the following day. The routine at our department is to perform IF for minimally displaced or undisplaced fracture (Garden I–II) FNF and hip arthroplasty for displaced (Garden III–IV) FNF in patients aged above 65 years. Total hip arthroplasty (THA) is used in the relatively young (up to 79 years) and active patients, in those with rheumatoid or osteoarthritic changes in the affected hip. Hemiarthroplasty (HA) is used in older (> 79) less active patients, those with low demands, those with short expected lifespan, and those with cognitive dysfunction. The final decision of whether to choose a THA or HA was made according to the surgeon’s preference and the patient’s level of activity. We included all eligible patients with a displaced FNF treated with a hip arthroplasty using a direct lateral approach.

IF was performed with the patient on a fracture table and the fracture visualized with an image intensifier and was fixed with two cannulated screws (Olmed; DePuy/Johnson & Johnson, Sollentuna, Sweden). In the anteroposterior projection, the distal screw was aimed at the level of the lesser trochanter to rest on the medial inferior cortex of the femoral neck. The proximal screw was positioned parallel to and at least 1 cm from the distal screw. Low-molecular-weight heparin was administered for 10–30 days postoperatively. Antibiotic prophylaxis was given on the day of surgery.

A cemented HA or THA was used through a direct lateral approach in the lateral decubitus position according to Hardinge. The HA was performed using the cemented SP II Lubinus system with a modular unipolar (Link® unipolar head, Warsaw, Germany) or bipolar head (Vario cup, Link®, Warsaw, Germany). The acetabular components were either a cemented acetabular cup (Link® Lubinus® Hip Acetabular Cup, Warsaw, Germany) or a cemented dual-mobility cup (Avantage®, Biomet, Valence, France) based on the preference of the treating surgeon. All patients with a displaced FNF treated with a hip arthroplasty through a direct lateral approach during the inclusion period were included. Antibiotic-loaded bone cement was used for all patients (Optipac®, Biomet, Sweden). Prophylactic antibiotics were administered 30 min preoperatively and 2 more times over 24 h postoperatively. Low-molecular-weight heparin was administered for 10–30 days postoperatively. All patients were mobilized during the first postoperative day with full weight-bearing according to a standard physiotherapeutic program without any restrictions.

### Data collection and follow-up

Using the unique Swedish personal identification number, we collected data by a combination of a search of the in-hospital medical database and follow-up visits. All patients were followed up until 2017 or until death. The minimum follow-up time was 22 months. We collected patient data including age, sex, ASA score, cognitive status, type of surgical treatment, and length of operation. The reoperation rate and mortality during the study period were also documented. An independent research nurse performed follow-up interviews 1 year postoperatively using the following patient-reported outcome measurements: Harris Hip Score (HHS), Western Ontario and McMaster Universities Arthritis (WOMAC) questionnaires, and pain numeric rating scale (PNRS) [11–14]. Data will be available on request from the corresponding author.

### Statistics

Student’s *t* tests and chi-square tests were used for continuous normal and ordinal data, respectively. All tests were 2-sided. For the PROM variables HHS, WOMAC, and PNRS, we used a generalized linear regression model to detect the differences between the 2 groups. Logistic regression was performed in order to evaluate the factors associated with reoperations. The study design and patients’ follow-up ensure non-informative censoring. The mortality during the study period was high and might therefore have affected the results obtained and decrease the initial power. A multivariable model adjusted for surgical treatment, age, sex, cognitive status, and ASA category (1–2 or 3–4) was included in the analysis. The generalized linear model was used because neither WOMAC nor HHS was normally distributed. Gamma distribution was used due to the right skewness of the curves. The associations are presented as odds ratios (OR) with 95% confidence intervals (CI). In each of the analyses made, there were 5 covariates. If 10–20 patients were required for each covariate included in the analysis, then the number of patients would be sufficient. Kaplan-Meier survival analysis was used to compare 1- and 2-year mortality between the groups. Statistical analysis was performed using SPSS® (IBM SPSS Statistics for Macintosh, Version 22.0, IBM Corp, Armonk, NY, USA). Trial registration: ClinicalTrials.gov (identifier: NCT03392285).

## Results

### Study subjects and descriptive data

**Fig. 1:**
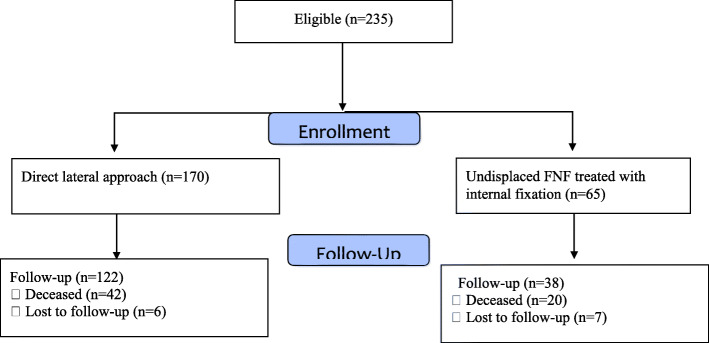
Flowchart

A total of 235 patients were included in the study with a median age of 83 years (range 65–99) (Table 1). Seven patients sustained bilateral FNF, and only the first fracture was included in the analysis. Sixty-five patients with undisplaced FNF were treated with IF, and 170 patients with displaced FNF were treated with hip arthroplasty (152 received HA and 18 THA). Figure 1 shows the flow of patients through the study. The baseline characteristics of the study groups are presented in Table 1 and showed no statistical differences between them. The median follow-up time was 26 months (range 0–56 months). The minimum follow-up time was 22 months. The mean length of operation was 35 min in the IF group and 90 min in the hip arthroplasty group.

**Table 1:** Study population characteristics. Continuous variables are presented as mean and range

	Internal fixation *n* = 65	Hip arthroplasty *n* = 170
Age	83 (61–98)	83 (64–99)
Sex		
Male	21 (32%)	54 (32%)
Female	44 (68%)	116 (68%)
Side		
Right	27 (42%)	77 (45%)
Left	38 (58%)	93 (55%)
Cognitive status		
Lucid	36 (55%)	100 (59%)
Impairment	29 (45%)	62 (36%)
Missing	0 (0%)	8 (5%)
Surgical treatment		
Hemiarthroplasty	0 (0%)	152 (89%)
Total hip arthroplasty	0 (0%)	18 (11%)
Internal fixation	65 (100%)	0 (0%)
ASA		
1–2	33 (51%)	73 (43%)
3–4	29 (45%)	94 (55%)
Missing	3 (4%)	3 (2%)

### Reoperation

Nineteen (8.0%) hips required reoperation at least once at a mean of 6 months (range 0–35) postoperatively (Table 2). All reoperations were performed during the first 20 months except for 1 periprosthetic femoral fracture that occurred 35 months postoperatively. The rate of reoperation was higher in the IF group compared to the arthroplasty group (13.4% vs. 5.9%, 0.9–6.4) (Table 3). The most common reasons for reoperations in the IF group were non-union/avascular necrosis where 6 patients were reoperated with hip arthroplasty (THA *n* = 2, HA *n* = 3) or an excision arthroplasty (*n* = 1). Three patients were reoperated with the removal of screws. In the arthroplasty group, the most common indication for reoperation was deep infection (3.5%) (Table 2). Patients with deep infection were treated with debridement, antibiotics, irrigation, and implant retention without any further reoperations. Six patients (3.6%) in the arthroplasty group had dislocation in whom 4 patients (2.4%) treated with closed reduction and 2 patients (1.2%) needed open surgical reduction.

**Table 2:** Reoperations presented as number of patients and percentage

	Internal fixation *n* = 65	Hip arthroplasty (*n* = 170)
Mechanical failure/non-union/AVN	6 (9.2%)	0 (0%)
Extraction of osteosynthesis	3 (4.6%)	0 (0%)
Deep infection	0 (0%)	6 (3.5%)
Revision due to dislocation	0 (0%)	2 (1.1%)
Acetabular erosion	0 (0%)	1 (0.6%)
Periprosthetic fracture	0 (0%)	1 (0.6%)

**Table 3:** Logistic regression presenting adjusted odds ratio (OR) for reoperations with 95% confidence interval (95% CI)

Variable	Reoperation	
	OR	95% CI
Cognitive impairment		
No	1.0	–
Yes	1.1	0.4–3.0
Surgical treatment		
Hip arthroplasty	1.0	–
Internal fixation	2.3	0.9–6.4
Age	1.0	0.9–1.1
Sex		
Male	1.0	–
Female	0.9	0.3–2.6
ASA		
1–2	1.0	–
3–4	2.1	0.7–6.2

### Patient-reported outcome

We did not find any differences between the two groups in terms of HHS (IF 67 vs. arthroplasty 70), WOMAC (79 vs. 80), and PNRS (2.7 vs. 2.1) (Table 3), also when adjusting for confounders: age, sex, ASA category (1–2, 3–4), and cognitive status (Table 4). There was a tendency towards a higher level of pain (PNRS) in the IF group (2.7 vs. 2.0).

**Table 4:** Patient-reported outcome variables. Generalized linear model regression including adjusted variables for Harris Hip Score (HHS) and WOMAC with 95% confidence interval (95% CI). Estimated marginal means (EM) for each covariate are included

Variable	EM	WOMAC		EM	HHS	
		Coef	95% CI		Coef	95% CI
Cognitive dysfunction						
Yes	77	–	–	67	–	–
No	81	4	− 4–11	69	2	− 4–8
Surgical treatment						
Hip arthroplasty	78	–	–	70	–	–
Internal fixation	79	− 0.5	− 9–8	66	− 4	− 2–11
Age	–	− 0.1	− 0.7–04	–	− 0.5	− 0.9–0.1
Sex						
Female	82	–	–	69	–	–
Male	75	− 7	− 14–1	66	–3	− 9–3
ASA						
1–2	80	–	–	70	–	–
3–4	77	− 3	− 4–10	66	− 4	− 2–10

### Mortality

The overall 1-year mortality was 28% (65 of 235 patients). The overall 2-year mortality was 40% (95 of 235 patients). Neither 1-year mortality (IF 31% vs. arthroplasty 27%) nor 2-year mortality (IF 45% vs. arthroplasty 39%) differed between the groups.

## Discussion

In this feasibility study, we did not find any significant differences in patient-reported outcome measures between the two groups although there was a tendency that patients with undisplaced or minimally displaced FNF treated with IF had more pain and a higher rate of reoperations.

Recent studies indicate that by using either a modern modular THA or HA, performed by a direct lateral approach, the risk for revision due to dislocation may be reduced to 2% [15]. The risk of periprosthetic femoral fractures is reduced to below 1% by the use of cemented composite-beam femoral implants [16, 17]. Periprosthetic joint infection poses a major challenge but might be reduced with the use of high-dose dual-impregnated antibiotic-laden cement to approximately 1–2% [18]. These improvements raise the question of whether arthroplasty might improve the outcome of undisplaced or minimally displaced FNF in the elderly [2]. There are limited data comparing IF and hip arthroplasty for these fractures, and trials have been called for to optimize the surgical treatment [2].

Reoperation rates ranging from 8 to 19% have been reported for undisplaced or minimally displaced FNF treated with IF [3]. In the present study, we found a reoperation rate of 14%, which is in concordance with previous studies [2]. The cumulative incidence of reoperation after IF for undisplaced or minimally displaced FNF was double that of arthroplasty for displaced FNF. There are a number of studies that have sought to predict the failure of undisplaced or minimally displaced FNF treated with IF. Posterior and ventral tilt on the lateral radiograph has been found to be a predictor of failure [19, 20]. Other risk factors include advanced age, nutritional status, and capital impaction [21, 22].

The patient-reported outcomes after treatment with IF for undisplaced FNF have only been sparsely reported [2]. Rogmark et al. found that 40% of patients reported pain when walking and 25% had pain at rest [5]. Despite uneventful healing of the undisplaced fracture, shortening of the femoral neck has been proposed as affecting postoperative hip function [23]. Elderly patients with reduced bone stock may be more susceptible for femoral neck shortening, mechanical failure, non-union, and avascular necrosis when treated with IF.

A recently published randomized controlled trial comparing hemiarthroplasty with IF for undisplaced FNFs found similar hip function measured by the Harris Hip Score [6]. However, regarding secondary outcomes, hemiarthroplasty led to improved mobility and fewer major reoperations.

Several factors affect the 1-year mortality such as age, cognitive impairment, pre-fracture mobility, and habitat. In the present study, we found an overall 1-year mortality rate of 28% with no significant differences between the two groups at 1 and 2 years. Hip arthroplasty might provide most elderly patients with a definitive treatment, equipped with a low risk for reoperation that allows immediate unrestricted mobilization without increasing mortality.

Our results could be used as a pilot study and guidance in the set-up of a randomized controlled trial comparing hip arthroplasty and IF in the treatment of undisplaced or minimally displaced FNF [7]. Conducting register-based randomized controlled trials, which include a randomization module in a large, clinical register with unselected consecutive enrolment, can combine important features of a prospective randomized trial with the inclusiveness and efficiency of a large-scale clinical register [24–26]. A recently published meta-analysis on the treatment of undisplaced and minimally displaced FNF, based on a total of 579 randomized patients, concluded that hip arthroplasty might reduce the need for revision surgery [27].

This study has limitations including the non-randomized observational design. First, our sample is powered to test the study hypothesis but is not large enough to detect smaller differences between the two groups. Second, the used outcome measure HHS have some disadvantages such as ceiling and floor effects, which could mask the small difference in patients who scored their status as very high or very low. Finally, the choice of treatment modality was chosen by the treating surgeon, and this might have contributed some bias to the validity of the treatment method in some patients. These limitations are counterbalanced by the strengths of the study, which is a prospectively followed cohort with minimal drop-out and adequate follow-up period. By using the unique Swedish personal ID number, we collected data by a combination of a search of our in-hospital surgical and medical database and follow-up visits which ensured a high data accuracy.

## Conclusion

In this feasibility study, we found no differences in patient-reported outcomes between the studied groups, although IF required a higher rate of reoperations and had postoperative residual hip pain. Further randomized trials are needed to establish the optimal treatment of undisplaced FNF in the elderly.

## Data Availability

The datasets used and/or analyzed during the current study are available from the corresponding author on reasonable request.
